# Collaborating in the context of co-location: a grounded theory study

**DOI:** 10.1186/s12875-016-0427-x

**Published:** 2016-03-10

**Authors:** Pamela Wener, Roberta L. Woodgate

**Affiliations:** Department of Occupational Therapy, College of Rehabilitation Sciences, University of Manitoba, R125-771 McDermot Ave., Winnipeg, MB R3E 0T6 Canada; College of Nursing, University of Manitoba, 465 Helen Glass Centre, 89 Curry Place, Winnipeg, MB R3T 2N2 Canada

## Abstract

**Background:**

Most individuals with mental health concerns seek care from their primary care provider, who may lack comfort, knowledge, and time to provide care. Interprofessional collaboration between providers improves access to primary mental health services and increases primary care providers’ comfort offering these services. Building and sustaining interprofessional relationships is foundational to collaborative practice in primary care settings. However, little is known about the relationship building process within these collaborative relationships. The purpose of this grounded theory study was to gain a theoretical understanding of the interprofessional collaborative relationship-building process to guide health care providers and leaders as they integrate mental health services into primary care settings.

**Methods:**

Forty primary and mental health care providers completed a demographic questionnaire and participated in either an individual or group interview. Interviews were audio-recorded and transcribed verbatim. Transcripts were reviewed several times and then individually coded. Codes were reviewed and similar codes were collapsed to form categories using using constant comparison. All codes and categories were discussed amongst the researchers and the final categories and core category was agreed upon using constant comparison and consensus.

**Results:**

A four-stage developmental interprofessional collaborative relationship-building model explained the emergent core category of Collaboration in the Context of Co-location. The four stages included 1) Looking for Help, 2) Initiating Co-location, 3) Fitting-in, and 4) Growing Reciprocity. A patient-focus and communication strategies were essential processes throughout the interprofessional collaborative relationship-building process.

**Conclusions:**

Building interprofessional collaborative relationships amongst health care providers are essential to delivering mental health services in primary care settings. This developmental model describes the process of how these relationships are co-created and supported by the health care region. Furthermore, the model emphasizes that all providers must develop and sustain a patient-focus and communication strategies that are flexible. Applying this model, health care providers can guide the creation and sustainability of primary care interprofessional collaborative relationships. Moreover, this model may guide health care leaders and policy makers as they initiate interprofessional collaborative practice in other health care settings.

## Background

Individual Canadians seeking mental health services are most often seen by their primary care provider (PCP). Watson et al. reported that 30–40 % of Canadians who visit their PCP have symptoms of a mental illness [1]. Individuals with mental illness make up at least 20 % of primary care patient visits [2] and take up approximately 25–50 % of the PCP’s practice time [3]. PCPs treat more than 50 % of Canadians who are seeking mental health services [4–6], while mental health specialists treat only 25 % of these individuals [4, 7]. Given these statistics, PCPs make a significant contribution to the overall Canadian mental health system.

Although PCPs provide most of the mental health services, their knowledge, skills, and comfort working with those who have mental illness varies. Some authors discuss family physician’s (FP) feelings of discomfort working with patients with depression [8–11]. Other authors, discuss the lack of PCPs’ knowledge and experience as a barrier to treating patients with depression. For example, Henke et al. describe a qualitative study using semi-structured interviews to gather information about the barriers to working with patients with depression. These authors collected data from 23 FPs who are practicing throughout the United States. In describing the study, the authors include their methods for creating the interview guide, the interview process and details of how they used a grounded theory approach to analyze the data. These authors reported six barriers to working with patients with depression including, difficulty diagnosing and a lack of experience. Anthony et al. conducted a mixed methods study of 40 PCPs including FPs, NPs, and general internists from one large urban centre in the United States [10]. These authors sought to understand PCPs’ decision to refer patients for depression care. The authors provide a thorough description of study process including, methodology, data collection instruments, and the specifics of the data analysis. The reported results of this study described the participants discomfort treating patients with depression. Prescribing medication is an important aspect of evidence-based treatment for depression and anxiety [12]. However, FPs report moderate levels of comfort prescribing medications for these patients [13]. For example, Craven and Bland [14] who conducted a comprehensive literature review reported that PCPs are comfortable treating individuals with mental illness who are responsive to medication that the provider is familiar with prescribing. Goossen et al. conducted a mixed methods evaluation of an existing CMHC program reported that PCPs, are less comfortable when medications need to be changed or combined [15]; a practice outlined in Canadian practice guidelines as an important part of improving a patient response [16].

In addition to prescribing medications, PCPs are aware of the effectiveness of evidence-based counseling. Grenier et al. surveyed 118 FPs in one Canadian province and found that 95 % of FPs knew of evidence-based counseling for depression and anxiety such as, cognitive behavioural or interpersonal therapy [17]. These authors note that a lack of time and training make it difficult for PCPs to implement counseling within their practices [17]. While individuals with mental illness are most likely to be treated by a PCP, the practitioner may not possess the comfort, training or time to implement evidence-based treatment, leaving patients with less than optimal mental health services.

PCPs believe that their ability to deliver mental health services would improve if they had support from mental health specialists [18, 19]. Acknowledging that most of the mental health services in Canada are provided by PCPs, physician leaders recognized the need to increase PCPs’ access to mental health specialists in primary care settings. In 1997, the Canadian Psychiatric Association and the College of Family Physicians of Canada together developed a position paper calling on PCPs and psychiatrists to work together [20]. In this paper, Kates, et al. declared that primary and mental health care providers were joining together to improve access to mental health services in what is referred to as shared or collaborative mental health care (CMHC), two terms that are used synonymously in this paper [14]. Furthermore, these two professional groups agreed that:

*family physicians and psychiatrists work more cooperatively to integrate their respective skills and expertise in a complementary and cost effective manner* ([21], p 1785).

Although it was agreed that generalists, PCPs and specialists, mental health providers would work together, little was known about how to develop the collaborative relationship and the importance of relationship building to the overall interprofessional collaborative process.

Today, well over 100 CMHC programs exist in Canada, each reporting successes [22]. For example, Kates discussed CMHC that were integrated into Ontario’s family health teams and who saw symptom reduction and improved functionality for 50 % of the patients with mental health concerns [23]. Bower et al. examined outcomes of CMHC for depression and concluded that partnering with case managers who receive supervision from a mental health specialist improved outcomes [24]. In terms of system changes, researchers report that CMHC results in increased access to timely psychiatric care [25–29], decreased referrals to outpatient psychiatry clinics [27], earlier detection of mental illness, reduced utilization of specialized mental health services [30, 31], and increased continuity of care [27, 28, 32, 33]. Researchers also found that individuals who participated in a CMHC program reported decreases in symptomatology, [32, 34–41]; less interference with social activities [32, 33], and increased satisfaction [28]. Furthermore, researchers report that implementation of CMHC increases PCPs’ capacity to work with individuals with mental illness. Several researchers found that subsequent to the initiation of CMHC, PCPs reported having increased, mental health care skills and comfort [27–29, 34, 42, 43], provider satisfaction [27, 34], and physician perceived patient satisfaction [34]. The World Health Organization (WHO) and the World Organizations of Family Doctors (Wonca) released *Integrating Mental Health into Primary Care* to justify the need to integrate mental health services into primary care settings. One of the key messages reported in this document is that there is less stigma and discrimination when patients with mental illness are seen in PC settings [44].

While there seems to be some agreement about the value of CMHC for individuals diagnosed with common mental illness such as depression and/or anxiety, there is little consensus about the patient outcomes of CMHC with individuals with serious mental illness. Fitzpatrick, et al. reported that CMHC did not improve patient outcomes for those individuals with serious mental illness [45]. Brown, et al. found FPs offered those with serious mental illness continuity of care, comfort and familiarity, and a whole person clinical approach [25]. In a chart review, Doey, et al. found that individuals with moderate to serious mental illness who participated in CMHC had reduced number of hospital and emergency room visits and patients reported high levels of satisfaction and continuity of care [46]. Smith et al. explored the effectiveness of collaborative care and found that while there is some reported improvements in patients with depression, the consistent finding was improved PCP prescribing practices [47].

Among those studying CMHC, there is some consensus about the components that contribute to an effective treatment program [48]. For example, most CMHC programs include a case manager; psychiatric consultation; brief forms of psychotherapy or counseling such as, cognitive behavioural approaches, motivational interviewing or interpersonal approaches; patient education; access to resources; and screening for depression and anxiety [48]. While these program components are essential, they must be developed upon an understanding of the PCP’s need for collaboration with the mental health specialist [15, 48] and a strong collaborative interprofessional relationship [25, 49–51].

Historically, PCPs and mental health providers report they have poor interprofessional relationships and a lack of mutual trust and respect [52] that seems to underpin a proclivity toward poor communication [19]. Kates stated that in addition to not meeting the needs of patients’ with mental illness, the relationship between PCPs and psychiatrists was poor including, insufficient access, poor communication, and a lack of understanding and support for the role of PCPs in delivering mental health services [20]. However after over a decade of CMHC, the Joint Working Group on Shared Care reported on the strides made in offering increased access to mental health services [48]. More recently, Goossen et al. [15] and Benzer et al. [11] recognized and reported that the interprofessional relationship is integral to shared care between primary care generalists and mental health care specialists. Although CMHC has been in place since the late 1990s, the development and sustainment of the interprofessional collaborative relationship aspect of the shared care model, has not been well developed. Thus while the shared care model has been widely implemented, we have little knowledge about how generalist and specialists build and maintain their interprofessional collaborative relationship. An increased understanding of how to build and maintain interprofessional collaborative relationships will provide much needed guidance to those health care providers attempting to navigate this complex process.

To date, there is little understanding of the relationship building process providers use to support the ongoing engagement to work together to provide primary mental health services. Understanding the providers’ perspective is essential to developing best practices that will ensure patients with mental illness receive the full benefits of the interprofessional primary mental health care team. Accordingly, we used a qualitative approach to explore the following study question: *How do primary care providers and mental heath care providers collaborate to provide mental health care in primary care settings.* More specifically the research objectives included:

1. *To detail the need for IPC in the delivery of mental health services in primary care from the perspective of the primary healthcare providers.*
2. *To detail primary healthcare providers and mental healthcare providers experiences and perspectives of IPC in the context of a primary care program, Collaborative Mental Health Care program.*
3. *To identify how the individual, group dynamics and system influence the IPC process in the context of the Shared Mental Health Care program.*
4. *To identify the opportunities and challenges of IPC in the context of the Shared Mental Health Care program*.

This paper describes the grounded theory of interprofessional collaborative relationship building that providers described developing and maintaining to deliver mental health services in PC settings.

## Methods

### Study design

This study was best approached from a qualitative research paradigm where the exploration is grounded in the providers’ experiences of IPC [53]. The purpose of the study was not to deduce a single truth, but rather to understand the multiple realities of the participating health care providers from an emic perspective [54]. More specifically, social constructivist grounded theory methodology [55] was used to facilitate an inductive exploration of the interprofessional collaborative relationship building process providers use to work together to deliver mental health services in primary care. Grounded theory as described by Charmaz is an appropriate methodology to use when the study purpose is to understand, rather than try to explain process. Social constructivist grounded theory acknowledges the co-creation of the study findings by both the researchers and participants [55].

Symbolic Interaction (SI) served as the guiding theoretical framework for this study. As SI focuses on the meaning individuals ascribe to an interaction, this framework helps us to explore multiple realities rather than to seek a single explanation [56]. In this study, using an SI lens, we focused on understanding the meaning provider participants ascribed to the interprofessional collaborative relationship building process as they engaged to provide mental health services in primary care settings. As SI focuses on meaning ascribed by individuals as they interact with other it is thought to be a useful framework when one is exploring process and change [55]. Further description of the study design and conceptual framework used is available in the methodology paper by Wener and Woodgate [57].

### Ethics, consent, and permission

The University of Manitoba Health Research Ethics Board provided ethical approval for this study (H2011:003). Informed consent was obtained from participants prior to the commencement of all interviews.

### Consent to publish

Consent to publish anonymized individual participant’s data was obtained as part of the informed consent process.

### Participants

Purposive sampling was used to recruit providers who participate in one health region’s CMHC service. All 110 PCPs, (100 FPs and ten nurse practitioners (NPs), 16 shared care counsellors, and eight shared care psychiatrists who participate in the health region CMHC program were invited to participate through recruitment flyers. We sought to achieve diversity in terms of geographical location of practice, physician remuneration model, and practitioner’s gender in the sample through maximum variation sampling [58]. There are 11 identified communities within the urban centre, seven of which have a CMHC service. Recruitment occurred from all seven communities that offered CMHC. In general, family physicians within this urban centre are remunerated using a fee-for-service model or receive a yearly salary. We sought to ensure that we recruited a relatively equal number of family physicians from each of the remuneration models. Previous studies have shown that the average socioeconomic status, education and health care needs vary among these communities (MCHP). We assumed that the patients living in each of theses communities are most apt to attend health care practices located within their communities and that these differences in income, education and health care needs, may contribute to the health providers’ interprofessional collaboration experience. Literature suggests that females are more apt to collaborate than males, therefore we attempted to ensure that we had representation of both male and female FPs, NPs, psychiatrists and counsellors [59, 60]. Sampling continued until categories could account for new data and *theoretical sufficiency* was achieved ([61], p 117).

### Data collection

Demographic information was collected to obtain a profile of the participants. Information about how the providers collaborate to provide mental health services in primary care was gathered using semi-structured in-depth individual interviews and focus groups that took place in a private room in the participant’s place of work. Data was collected from three groups of participants: 1) PCPs, 2) groups of providers that included FPs, NPs, psychiatrists, and counsellors, and 3) health authority regional leaders. First, PCPs were interviewed individually. The initial interview guide was created based on the results of a literature review and a previously completed program evaluation [15]. The interview guide for the individual PCP interviews included open-ended questions about the patient population served, experiences providing mental health services, need for collaboration with mental health specialist and their experiences of collaboration.

Second, interprofessional focus group interviews including PCPs, and mental health care providers were conducted. The focus group interview guide was based on the data analysis of the PCP interviews and the literature, and focused on understanding the details of the providers’ experiences of interprofessional collaboration to provide mental health services to patients. Focus group interview questions were created based on the emergent themes from the PCP individual interviews and the literature, and included asking providers about the meaning of interprofessional collaboration, process of collaborating, strengths and challenges of interprofessional collaboration, process of resolving conflicts among team members, influence of co-location on the interprofessional collaboration process, and the role of the health region in interprofessional collaboration. Questions about interprofessional conflict were added to the interview guide when it was noticed that participants did not discuss this issue, although it is reported in the literature. Third, interviews with the regional leaders were conducted. The Regional leaders’ and decision-makers’ interview guide was created based on the emergent findings from the previous interviews. Although these interview guides were used for all interviews, the interviewer (PW) was responsive to participants’ inviting them to further discuss issues raised. As well, the interviewer encouraged the participants to raise any issues that the participants wanted to discuss prior to ending each interview. A sample of interview questions from all three guides is included in Table 1.

**Table 1 PCP Individual Interview Sample Questions:** ᅟ

1. Tell me about your primary care practice?	
2. Describe the patient population in your primary care practice?	
3. Tell me about your experiences in your practice of providing health services to patients with mental health problems?	
4. Tell me about an experience where you were asked by a patient to provide mental health services/support to a patient when you felt comfortable or equipped to do so?	
5. Tell me about an experience where you were asked to provide mental health services/support to a patient when you did not feel comfortable or equipped to do so?	
6. What have been your experiences working with the psychiatrist?	
7. What have been your experiences working with the counsellor?	
8. What kinds of decisions were made during these collaboration?	
9. How did the collaborative decisions meet your needs?	
10. How did the collaborative decisions meet your patient’s needs?	
PCPs and MHPs Focus Group with Sample Questions:	
1. Tell me what Interprofessional collaboration means to you?	
2. In your particular practice tell me who is involved in the interprofessional collaboration process to deliver mental health service?	
3. How does co-location influence the interprofessional collaboration process?	
4. Tell me about your approach to patients?	
5. How is information such as decisions communicated between health care providers?	
6. What are your team’s strengths?	
7. What have been your biggest challenges collaborating to deliver mental health services?	
8. Tell me what happens when there is disagreement between providers? How are conflicts resolved?	
9. How does the Shared Care program or the WRHA support interprofessional collaboration to deliver mental health services?	
Regional Leaders and Decision-makers Focus Group and Individual Interviews Sample Questions:	
1. From your perspective, what is the role of the various team members in delivering mental health care?	
2. What do you see as your role in relation to delivery mental health care in primary care settings?	
3. Shared care is thought to involve interprofessional collaboration, what does that mean to you?	
4. Describe how interprofessional collaboration is used to deliver mental health services in primary care?	
5. What structures does the program or the region provide that supports interprofessional collaboration in Shared Care Mental Health? Are there other structures that you think would provide additional support or facilitate greater collaboration?	
6. What processes do you think are facilitative of interprofessional collaboration and how does the program or region support these processes? Are there other processes that you think could make a facilitating contribution to interprofessional collaboration?	
7. Describe any or how the program or region impede interprofessional collaboration? What kinds of things could be changed to remove these barriers?	
8. What role does this group play in developing and facilitating interprofessional collaboration?	
9. What resources does this group access to encourage and support interprofessional collaboration? What kinds of resources are missing/unavailable that could further support interprofessional collaboration?	

### Data analysis

All demographic questionnaires were analyzed using descriptive statistics. Individual and group interviews were audio recorded and transcribed verbatim. Prior to initiating coding, the transcripts were read several times to gain an understanding of the whole. In keeping with grounded theory, the coding process consisted of initial and focused coding phases [55]. We analyzed the data, assigning initial codes for each transcript and writing memos to form initial definitions [55]. Using focused codes as preliminary categories, we wrote more in-depth memos from the first seven interviews and used constant comparison, remaining open to new and emerging categories as we analyzed the remaining interviews [62]. Authors met to discuss the overarching theme and categories to achieve consensus. Interview transcripts and a newsletter describing the preliminary findings were mailed to all study participants for feedback prior to the finalization of the overarching theme, categories and developmental model however, no participants suggested changes to the proposed categories.

We included several methods to ensure study rigour [63]. The credibility and dependability of this study was established by aligning data collection methods with the study questions [57]. Data was collected over a long period of time and included participants from different geographical locations and from practices with different remuneration models. We kept an audit trail and reflexive journal to establish confirmability [63]. Transferability was explored by sharing the overarching theme, categories and developmental model with study participants and solicitation of feedback at conferences, presentations, and from peers [62–64].

## Results

### Description of participants

Health care providers (*n* = 32) and health region leaders (*n* = 8) participated in this study and completed the demographic questionnaire. Of the health care providers that participated in the study, there were 16 (50 %) FPs, 8 (25 %) nurse practitioners (NP), 3 (9.4 %) psychiatrists, and 5 (15.6 %) counsellors. Of the 16 FPs, 10 (62.5 %) reported that they participate in the provincial fee-for-service (FFS) remuneration program and 6 (37.5 %) of the FPs stated they receive a salary from the region (SFP). All NPs, psychiatrists and counsellors receive a yearly salary from the health authority, the regional body responsible for health care delivery.

The providers’ ages varied within each of the provider groups from 30 years to over 60 years of age. However, within the PCP sample, FFS FPs tended to be older than either the SFPs or the NPs and the NPs tended to be older than the SFPs. For example, 70 % of FFS FPs reported they were 50 years of age or older while none of the SFPs or NPs were over 50 years of age. In terms of years with the CMHC program only one of the 32 health care provider participants had been with the CMHC program for less than one year, while ten participants had greater than 5 years’ experience in the program. Taken together the health care providers worked in 12 different primary care clinics that varied in geographical location within the health region. Eleven FPs and five NPs participated in the initial individual interviews that took place over a 1 year period, March 2011 to February 2012. The six focus groups included 2–4 participants and took place over a 6 month from the end of November 2012 to May 2013. One counsellor, and two psychiatrists participated in more than one focus group interview because they provide service to more than one clinic. In these cases providers were directed to talk about their experiences in each clinic within the separate focus groups. One family physician participated in both an initial interview as well as a focus group and no specific directions were provided by the interviewer.

In addition to these health care providers, eight members of the regional leadership group participated in either a focus group or an individual interview based on the individual’s ability to attend the focus group. These interviews took place over a 2-month period from July 2013 to August 2013. The regional health leaders included individuals who belonged to a variety of health professions and had additional education in, health systems and administration. The members of this group were responsible for overall implementation and monitoring of the CMHC program. Pseudonyms are used in this manuscript to maintain confidentiality of study participants.

The findings revealed one overarching emergent theme, Collaborating in the Context of Co-location that includes a four-stage developmental interprofessional relationship building model. The emergent categories were the four stages of the developmental model and included: *Looking for Help, Initiating Co-location*, *Fitting-in,* and 4) *Growing Reciprocity.* This model and four developmental stages describe the role of the health region leaders and the providers in creating interprofessional relationships amongst the PCPs and mental health care providers. These relationships enabled providers to deliver primary mental health care. The authors used member checking to confirm that the developmental model and stages were an accurate representation of the participants’ interprofessional collaborative experiences. These developmental stages held true across professions and gender.

*Collaborating in the context of co-location* was the overarching theme that describes the evolving interprofessional relationships between primary care and mental health care providers for the purpose of meeting primary care patients’ mental health needs. Collaborating in the context of co-location is how the mental health care providers who are part of the CMHC program are situated within the PCPs office to facilitate the PCP’s patient-focused provision of primary mental health services. Lisa, a nurse practitioner describes how she and a co-located psychiatrist were able to provide mental health care when otherwise, this patient would not have received treatment. Furthermore, the psychiatrist is able to fulfill the NP’s patient care need, being available at the patient’s PC appointment time: *I can think of at least, well more than one time… I had someone that was clearly very ill, with no insight. And would not agree to come and see a psychiatrist. I needed that assessment done… I just had to arrange for him to have an appointment with me… and then have our psychiatrist just kind of join us… being co-located allowed for that to happen.* (NP, Lisa)

**Fig. 1:**
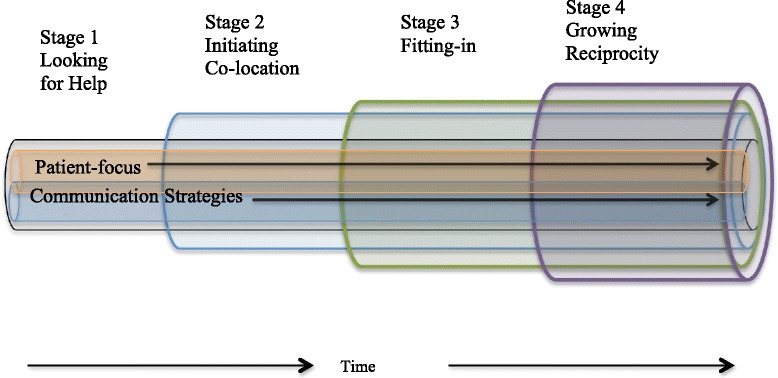
Stages of Interprofessional Collaborative Relationaship Building

In supporting PCPs, all providers use a variety of communication methods with the explicit intention of learning to work together to both provide and enhance the capacity of primary mental health services. The providers’ evolving relationship proceeds through four stages over time that begin with looking for help to provide mental health services, to a stage where providers participate as partners of patient care as shown in Fig. 1. During each stage of development the providers build upon the aspects of the relationship established during the previous stage. The groups of providers were always focused on patient care using varied communication strategies that were implemented flexibly depending on the needs of the individual practice. Overall, co-located groups of providers moved through the stages at different rates of time and not all interprofessional collaborations develop to the stage of growing reciprocity.

#### Stage 1-looking for help

*Looking for help* is when the PCPs and regional leaders look to mental health experts to work with PCPs to help PCPs to deliver mental health services in their primary care settings. Participants in this study expressed their need for help; access to mental health services and clinical experts to help them increase their mental health knowledge and skills. PCPs in this study, discuss how they need timely access to mental health services and how this access was not available prior to participating in CMHC program.

*I have worked at other places where a 3-month wait for psychiatry and an eight-week wait for counselling is a short wait. Usually by that time, the problem that the person has come in to ask for help has now fizzled in one way or the other. So you’ve missed that opportunity. So access in a timely manner is massive. And I think that that only expedites the patient’s ability to improve or get better.* (NP, Evelyn)

Although PCPs are patient-focused and want to provide mental health services to primary care patients, they perceive they have a lack of time, comfort and/or expertise. Comfort working with patients with mild to moderate mental illness varied amongst the PCPs participating in this study, with more experienced PCPs reporting that their comfort working with patients with a mental illness has grown over time and with life experiences. Sarah expressed this growing comfort:

*I think as a whole with being in practice for a long time…I think part of it is just my own experience and my own competence or comfort with feeling not as overwhelmed with some of the people that come in with those problems.* (FP, Sarah)

Participants in this study all reported that patients with mental illness that are difficult to diagnose, or that have a personality disorder, and those that are not responsive to medications require that PCPs have specialized knowledge and skills that are beyond their own clinical capacity. For example, this FP with many years in clinical practice describes the circumstances when he requires specialist help. *…mild to moderate depression I can usually handle. People with severe depression, people who present with mild to moderate depression who are not responding well to my initial approach, that’s where the call for help usually comes in* (FP, Gary). As patient-focused PCPs, these study participants want to provide primary mental health services, are aware of their knowledge and skill limitations, and require help from mental health specialists.

At a health region administrative and clinical level, the leaders identified and embraced the need to enhance mental health services in primary care settings through interprofessional collaboration between generalist PCPs, and specialists mental health care providers. As another regional health leader explained, the mental health service enhancement in primary care was logical as PCPs were already playing a key role in the mental health system, *…the need for collaboration… primary care physicians are providing a significant amount of mental health services. That’s a driver.* (Regional Leader, Ralf)

#### Stage 2-initiating Co-location

*Initiating Co-location* is the regional leaders belief in the usefulness of the CMHC model and then situating the mental health providers into the primary care clinics. As this regional leader explains, learning about collaborative mental health programs from an expert convinced her that co-location of providers was the next step in improving the mental health system: *I had been to a conference with Nick Kates* (Canadian Founder of Collaborative Care) *and gone to a couple of presentations and thought, this* (co-locating providers) *is where we need to go as a system* (Regional Leader, Leanne)*.*

Initiating co-location, that is, geographically bringing providers together signaled to the PCPs and mental health care providers that the leadership was committed to intra- and interprofessional collaboration in primary care sites. As this counsellor and psychiatrist describe, creating the structures and processes to co-locate providers meant the regional leaders believed in the program: *the* (health) *region supports collaboration because they’ve put this structure into place for us* (Counsellor, Nofar); *they* (the health region) *pay me a salary that I’m able to participate in the program* (Psychiatrist, Eleni). Regional leaders secured the services of the psychiatrists, and counsellors providing yearly salary arrangements with an understanding that their days would include time for collaboration. Another counsellor and psychiatrist explain how initiating co-location, the regional leaders understand that providers need face-to-face time and value it as a critical component of the program. In this example the providers use the term *collaboration* to mean face-to-face time working together.

*…if I’m spending* (face-to-face) *time collaborating with any of the primary care providers, I know that Shared Care sees that as a legitimate use of my time. …from a Shared Care perspective, we still need to see a certain amount of people but the* (face-to-face) *time spent collaborating is equally or more important even than that as a program.* (Counsellor, Elia)

*It’s* (collaboration) *valued.* (Psychiatrist, Daniel)

Unlike the PCPs on salary, initiating shared care in FFS PC sites regional leadership needed to be more flexible in how and when providers were co-located. For example, regional leaders had to negotiate with providers about the use of rooms and time for collaboration. This FP describes how part of bringing the providers together meant that providers needed to be willing to provide space for the mental health providers. While this may initially be perceived as negative, financial compensation alleviated the situation:

*…it might actually work even a little negatively because Patty (counsellor) is using one of my rooms and if I have a resident then I’m short one room, but Shared Care does pay us sort of a token rent so in the long run there’s no negative* (FP, Hart)

#### Stage 3-fitting-in

Fitting-in is when co-located mental health providers and PCPs begin to interact within one another to provide mental health services to PC patients’. For many PCPs, bringing providers together was about creating a familiarity with the specialist provider that was profoundly different from the historical non-co-located generalist/specialist relationship. In this relationship, the mental health care providers work to fit-in into the PC clinics, interacting with the PCPs as they provide mental health services that the PCP identifies needing for the patients. During this stage all PCP study participants identify needing mental health consultation for diagnosis, medication management, and therapy. Essential to this this developmental stage is the mental health care provider being flexible with their time in order to fit in with the unique schedule of a PC clinic and/or the PCP. For example, one counsellor purposely altered his schedule to stay late into the early evening, ensuring that he was free to meet with the physicians when they were available. One of the psychiatrists at another PC setting describes waiting outside of physician’s examining room to be able to *catch the doc between appointments*. Another FP describes how the psychiatrist and counsellor have, *learned to fit with him, Because I don’t eat lunch downstairs. So, in my office, they’ve learned that, So if they want to find me they can.* (FP, Michael)

During this stage mental health care providers needed to develop patient-focused communication strategies that were flexible and fit with each PCP. However, in some practices psychiatrists and counsellors reported that not all PCPs consulted with them nor did all PCPs meet with them about the patients seen. Both mental health providers and PCPs described how fitting-in occurred with some providers and not others but in all cases mental health services were not provided and the relationships did not progress. This counsellor describes how she is able to develop a relationship with those PCPs willing to meet with her and the challenge when PCPs are not prepared to make the time to share in the care of patients:

*The challenges, that I believe that we get along really well but I can’t say that for every physician…And people do have different willingness to meet and to share and collaborate…. it’s like getting the mail delivered. They love having it come to the door and they don’t want it. But they don’t want to necessarily go to the corner to pick it up, you know. And so we’re here. Are they willing to put in extra effort? To work with me I would say, yea, it’s kind of a working collaboration. Not just the talking.* (Counsellor, Lori)

For this FP it is clear to him that when a PCP is not willing to meet with the mental health care provider then the PCP is declaring that they are opting out of the collaborative relationship.

*…if you’re providing a service for us and be willing to talk to us and everything else, to just say I won’t ever sit down with you and talk.…fine, then you’ve excluded yourself from this group…. It’s just got to be that way at some point.* (FP, Michael)

Using patient-focused communication strategies such as short hallway conversations or patient referral forms along with the mental health care providers’ timely service provision, providers become more familiar with one another and their interprofessional relationships develop. One of the FPs describes how the face-to-face patient-focused interaction between providers is a key aspect of creating familiarity: *we’ve said over and over again that’s been a huge part …you literally can talk to somebody in the hallway … just that physical presence is helpful … a huge part for us* (FP, Adi). Collaboration was difficult for PCPs who did not fit in with providers at particular clinics. For example, when the mental health care providers work on days when a PCP was not present, the PCPs did not perceive that the mental health specialist service was available:

*… maybe that is there (the ability to email or call the psychiatrist) and I’m just not aware of it. …I’m not in every day, she’s in on a day that I’m not here, …I don’t ever see her….* (FP, Jacquie)

Most of the mental health providers discussed how they expected PCPs to discuss their referral to the psychiatrist or counsellor with the patient to ensure there was an understanding and agreement from the patient. This counsellor suggests that PCPs who do not accept their responsibility do not fit with the CMHC program.

*I have someone* (PCP) *who habitually sends me people that don’t show up. That this person* (PCP) *kind of doesn’t get it or they don’t communicate to their patient what it’s really all about and why they have to come or why they would benefit by coming. I wouldn’t want anybody seeing me because they have to. Because as you’re, some people* (PCPs) *just won’t fit, you know. Because they have, there’s some responsibility to do something.* (Counsellor, Lori)

However, as Juliette describes during this third stage when providers fit in with the PC clinic, collaboration within the context of co-location moves beyond physical proximity of providers to the receptivity providers feel amongst them: *… the biggest difference is one of familiarity cause I see Samantha (the counselor) every day that I work here and Gretta (the psychiatrist) …she’s very approachable, she’s happy to talk about cases.* (NP, Juliette). As the providers work together to ensure the patients’ mental health needs are met, they are simultaneously creating interprofessional communication and service delivery strategies that work for their particular PC practice.

Written communication is an important aspect throughout *the fitting-in stage*. PCPs initiate a consultation to a mental health care provider and receive written consultation reports. While mental health services are provided to the patients, mental health care providers write progress notes in a common patient chart or electronic medical records (EMR). These written forms of communication contribute to building PCPs’ mental health knowledge, skills and comfort. FP participants describe how the specifics of the written communication processes are important to the PCP’s capacity to treat patients. This FP describes that because the written consultation includes treatment specifics, it is facilitative of the provider’s ability to comfortably treat the patient:

*I would look to that written consult… they’re very specific as far as recommendations go for medications, for doses, for resources,* (FP, Leslie)

In contrast, one FP describes the inconsistent communication she typically experienced prior to participating in the CMHC program:

*I had a patient who has a mood disorder who was admitted… I worry about these people when I don’t see them, a discharge summary may come four months after they’ve been discharged from hospital, the flow of communication is often lacking.* (FP, Adi)

Although the written forms of communication are important, once the mental health consultation process was initiated, the PCPs relied on talking directly to the mental health providers for day-to-day patient-focused service provision. As this nurse practitioner describes, talking with the mental health provider facilitates timely treatment planning that is perceived to be meeting the patient’s needs.

*…she was evaluated and then we had a conversation right at my desk, right after she was evaluated and we talked about what do.* (NP, Donna).

All participants discussed that during this fitting-in stage, being familiar with one another facilitated direct communication, such as *quick talks before a patient is seen or after a patient leaves the visit.* Most study participants describe using direct communication between the PCP and counsellor as an efficient and timely approach to patient care.

#### Stage 4- growing reciprocity

This last stage in the developing interprofessional collaborative relationships in the context of collaboration is when the providers come to know and care about one another, value each other’s personal and professional expertise, and discover shared patient care values. The PCPs in this study appreciated when the psychiatrist and shared care counsellor shared their knowledge and suggested assessment and treatment approaches that enabled the PCP to respond to patients mental health needs confidently and in a timely manner. PCPs who participated in this study expressed an unequivocal trust in the psychiatrist and shared care counsellor. For example, Jacquie a FP, expresses appreciation for and confidence in the medication management suggestions provided by the psychiatrists: *…if I’m having trouble getting the right medication, then I’ll refer to the psychiatrist and then I definitely take their opinion…*(FP, Jacquie)*.* Many study participants shared that they implemented the treatment recommendations as suggested and that they would not consider changing what was recommended: *…I would never alter it from what the psychiatrist has suggested but initially make sure I follow that exactly as they’ve suggested…* (NP, Susan). On the other hand, this FP defines the interprofessional relationship in terms of being most responsible and acting on behalf of the patient:

*I’m still quarterback, I’m still the guy that’s running the show for my patient and I’m ultimately responsible for what’s going to happen, and I have to take the advice of the consultant and decide whether I think this is appropriate or not…Sometimes knowing your patient or knowing a different circumstance saying this isn’t going to work you may not follow that bit.* (FP, Ira)

Participants also express relief and appreciation that the shared care counsellor knew of other mental health resources that the patients could access: *…knowing what other places offer counselling cause that's one of the big black holes out …I have a sense of a few things just that I’ve learned over time, but she* (counsellor*) knows a whole lot more than I do so.* (FP, Adi). The PCPs relief is coupled with the counsellors’ recognition of how their ability to provide assistance deepens the developing interprofessional relationship: *…once somebody sees you actually can be helpful that will go a long way in building a relationship*. (Counsellor, Brandon)

During this stage, the interprofessional collaborative relationship becomes deeper, as the valuing of one another process becomes reciprocal and providers recognize that they have shared values such as providing holistic patient care. This FP describes the psychiatrist or counsellor looking to him to ensure the specialist has a complete and holistic understanding of the patient*…they’ll call me in and ask if any other thoughts that I have* [sic]*, cause a lot of these people I’ve known them for 35 years, I have the advantage of experience with them.* (FP, Hart). Similarly, the mental health providers value and understand how the PCP’s long-time knowledge of the patient was an important aspect of patient care:

*There’s a lot of brainstorming too because if I just meet a client, for the first time, I’ll come back,* (to the PCP) *…these guys know that client well. And so I’ll say, well this is my impression or this is kind of my feeling, what do you think? And so then it’s usually we tease out kind of where we go together, you know.* (Psychiatrist, Eleni)

At this stage there is an ease and comfort between providers that has moved beyond a one-way valuing to a more comfortable reciprocal relationship that is based on a shared value of *providing patients with the best care possible*. As this provider describes there is an increasing comfort that includes flexibility *…sometimes I will go there or they will go here or we’ll meet in the corridor and say I’d like to talk about so and so and it’s a very comfortable relationship.* (FP, Gary) For some groups of providers, a perceived non-hierarchical structure was an important contributor to the growing reciprocity. This counsellor describes the impact of perceived non-hierarchy on the providers’ sense of cohesiveness

*there’s respect for the different roles that people play within the clinic…that has separated this clinic in terms of functioning and cohesiveness in a way that lots of clinics set up similarly haven’t really been able to achieve. And I think that it’s really been because of taking out that hierarchical structure. That has made the clinic function so much better as a workplace.* (Counsellor, Corey)

During this stage providers’ shared value of being patient focused is heightened and together they create relationships that ensure patients have timely access to mental health services, while at the same time, retaining the PCPs’ position as the key health care providers. This FP shares how the PCP and mental health specialist expressed their joint commitment to timely patient focused care:

*I know myself and at least one of my other colleagues may call him up and saying you know I’ve got this person or what do you think about this medication for this person that you already know and being able to make a lot of those decisions with his you know okay or with his input on a more informal and timely basis.* (FP, Adi)

PCPs describe developing relationships with mental health providers that are based on trust and respect, and how this creates not only trust between providers but also trust between PCPs and the patients. This provider describes how the patients benefits from the established relationship among providers:

*… from the patient’s perspective that’s helpful that we actually know each other. I’ve said to people there’s other specialists …I don’t know them but I think they’re good… I think from the point of the view of the patient because it’s very personal that everybody’s kind of connected.* (FP, Sarah)

Many of the study participants described that the collaborative relationship developed over time. This PCP share the sense of ease and trusting collaborative relationship that develops over time:

*It’s also about establishing a relationship with them as well…I think the more you collaborate, the more you understand each other and the more your thinking tends to line up around how you deal with your patients or your clients. Like working with [counsellor’s name] for 8 years, I know how [counsellor’s name] thinks. I know what her patients are like. I know how she is going to treat her patients. I’ve worked with [psychiatrist’s name] for, I don’t know* (FP, Jacquie).

Another FP describes how the collaboration facilitates patients receiving the right care at the right time:

…*if the counsellor, was to see somebody and thought this person needs medication, they would come out and talk to me about it or as I say if it’s somebody that I think really needs to be seen more quickly than average I will make a point of going around and talking to the counsellor…* (FP, Gary)

At this later stage of development the health care providers anticipate that as they come together to provide patient care, there will be different opinions about how best to meet the patient’s needs. Providers in this study understood that these differences emanate from the providers having different knowledge and skills but that all providers are motivated to do the best for the patient. Understanding that all providers share a common interest in meeting the needs of the patient seems to help the providers reframe interprofessional provider into a culture that welcomes diverse perspectives:

*The only times there has been somewhat of a difference has been more on the impressions that we’ve had of what’s going on because we come to it from two different angles. But I don’t think there’s ever been really a disagreement about how to go forward from there because it does always involve the patient and their opinion…, and their preferences. And it does also always come from a place of wanting to do the best that we can by that person. And so it’s hard to imagine conflict when you have the same ultimate goal in mind.* (Counsellor, Corey)

Providers express the evolving collaborative relationship with mental health providers as caring about one another on a more personal basis. This FP explains how when providers work together and get to know one another on a more personal basis, the relationship deepens and creates a closeness between providers that enriches the work relationship:

*…when you know somebody and you know that they’re due with their next pregnancy or who their husband is and you know what their kids do.. It’s really hard to have a bad relationship when you know people really well. And it’s so much easier to have great working relationships when you are that intimate with people…* (FP, Taryn)

## Discussion

Our study describes the stages of developing interprofessional collaborative relationships in a CMHC program in a primary care setting which to date, has received limited study. Using an SI lens allowed us to understand the meaning that the interactions between the regional leaders, PCPs, mental health care providers and the primary care context contributed to provider perceived interprofessional collaborative relationships. The results of our study situate co-location as a crucial component to developing interprofessional collaborative relationships in the shared care, primary care practice setting. Co-location has consistently been identified as an important factor in building collaborative teams between those in mental health and primary care [14, 41, 65]. Allport found that interpersonal contact is an effective way to overcome intergroup conflict, a suggestion he put forward as the *contact hypothesis* [66]. In this study, co-locating providers set the stage to develop interprofessional collaborative relationships. Similarly, Kates et al. reported that co-location enhances communication and eases the referral process, case discussions and improves continuity of care [27]. Participants in this study described that co-locating providers encourages interprofessional interaction that they perceive to be critical to the developing interprofessional relationships.

Hewstone and Brown agreed that interpersonal contact is important, however, they state that it is not sufficient to increase trust among group members [67]. These authors suggest that to increase trust among group members there also needs to be personal interaction, equal status, common goals, support from the institution or agency, and cooperation. Mulvale et al. found that personal contact and face-to-face case conferences between providers is an important contributor to the success of the CMHC program [41] and FPs who worked with co-located counsellors and psychiatrists reported the highest levels of satisfaction [18]. The participants in our study also emphasized the importance of both face-to-face interaction as well as written forms of communication. Providers in this study also discussed the importance of a non-hierarchical structure, a common focus on improving patients’ mental health, and support from the program and health region leadership.

In this study, participants from different practices described a similar road taken to develop their relationships that included co-location of providers, a focus on fitting-in to the PC culture and clinic, and then a sense of having arrived at a mutually respectful and collaborative relationship where providers knew each other professionally and personally. However, while this study describes the patterns of the interprofessional collaborative relationship development, it falls short of helping us to understand what and how the team propels itself forward.

While the stages of the interprofessional relationship building process in a CMHC program have not been described previously, Chidambaram and Bostrom conducted a review of group development models. These authors described two broad types of group development, sequential and non-sequential [68]. In health care, most authors describe team development using a sequential linear progressive model where the team matures and is defined by improved performance over time [69]. Tuckman and Tuckman and Jensen’s sequential lineal progressive model that includes five stages of development [70, 71] is widely accepted by experts of small group processes. Moreover, this team developmental theory has been used to describe interprofessional health care team development [72–74]. However, while the study participants described that interprofessional collaborative relationships develops over time, the participants in this study also describe the critical role of the regional leaders in the interprofessional team development.

In our model the regional leaders play an important role in the first two stages, identifying the need for interprofessional collaboration and initiating co-location of providers. Organizational leaders have long been recognized as an essential element to successful interprofessional collaboration. For example, San Martin-Rodriguez reviewed theoretical and empirical studies to determine the components for successful collaboration. These authors found that when the organization believes in interprofessional collaboration i.e. identify and/or understand the need for collaboration and create physical proximity between providers are among the important features necessary for interprofessional collaboration [75]. D’Amour and Oanadasan, 2005 also suggest that the organizational leaders or decision makers must be supportive and play an important role in implementing interprofessional collaboration [76].

The participants in this study describe *fitting-in,* where the mental health care provider fulfills the PCP’s patient needs by sharing their clinical expertise. As the PCPs recognize that their patient needs are being met, all providers begin to respect, trust and value one another, similar to the “norming” process that is Tuckman’s third stage of group development [70]. In a recent study, Benzer et al. reported that when mental health care providers in PC settings attend to the PCPs identified patient needs, communication between the PCPs and mental health care providers increased [11]. While Benzer’s work makes an important contribution to our understanding of interprofessional communication, it was not describing developmental stages nor grounded in health care providers’ experiences.

The fourth stage of our proposed relationship building model, *Growing Reciprocity* includes descriptions of increased cohesion, a sense of trust, belonging, and togetherness. Cohesion is reflected in the study participants’ discussion of comfort, trust, respect, sharing of values, and valuing of differences in opinion amongst providers. Cohesion, is thought to be an essential feature of group performance [77, 78] and was identified as a key component of interprofessionality [76, 79]. While several participants in this study discussed the importance of cohesion, further research would need to be done to understand the role of cohesion amongst the interprofessional health care providers.

In our study, participants discovered that they all valued a patient focus and holistic care that addressed patient and provider needs. As the participants in our study worked together, they recognized that they needed to be flexible depending on the primary care context and the unique needs of the patient and/or provider. Participants described adapting their communication strategies, approaches and schedules to meet each other’s and the patient’s needs.

The two central components of our model, communication strategies and the patient-centred approach have been reported findings of several previous studies. A commonly reported findings is the importance of providers communicating openly aiming towards reciprocal dialogue [18, 25, 32, 34–41, 46] while Lucena and Lesage, discuss the importance of written communication strategies [80]. In support of the second key finding, authors describe how a focus on the patient may assist teams in dealing with role conflict [25, 28, 81]. Team conflict is often a result of role boundaries, scope of practice, and accountability. However, in our study providers focused on providing patient focused care where the PCP requested interprofessional collaboration based on the patient’s identified need for mental health services. Rather than focusing on areas that are the typical sources of conflict, such as role boundaries and scope of practice [82], providers in our study recognized that consideration of all of the perspectives may best meet the patient’s needs. Maintaining a patient focus helped providers in our study to not categorize the varying opinions as “correct” or incorrect, rather they were understood as a reflection of various professional knowledge and expertise. The Canadian Interprofessional Health Collaborative established interprofessional communication and patient-centred care as foundational competencies for interprofessional collaboration [83]. Flattened hierarchy [81] and flexibility [49] have also been discussed in the shared care literature, although not conceptualized within a model that facilitates the interprofessional collaborative relationship building process.

Findings from our study make an initial contribution to our understanding of the developing interprofessional collaborative relationship between health care providers. More research is needed to understand how the components of the interprofessional collaborative relationships within a stage of development facilitate or impede team development. Future research may also explore the application of this interprofessional collaborative relationship building model to other practice settings. This collaborative relationship building model highlights co-location of providers; future research may explore virtual interprofessional collaborative teams and the processes they use to develop their relationships. Other limitations of this study include the possibility that only providers having positive interprofessional relationship building experiences volunteered to participate in this study thus, limiting our understanding of the role of conflict and conflict resolution. Furthermore, in this study the patient voice was represented by the health care providers and not by the patient themselves. Future research on the interprofessional collaborative relationships should include asking patients directly for their perspective [84].

## Conclusion

Increasingly health care providers are asked to work collaboratively with their colleagues from other professions. However, little attention has been given to how these professionals are to initiate and maintain these interprofessional relationships. Providers participating in CMHC programs within Canada, collaborate to successfully provide mental health services in primary care settings. Exploring and documenting how these providers develop and maintain their interprofessional collaborative relationships contributes to our overall understanding of the importance of the provider-to-provider relationship. Recognizing that relationships develop in stages and require time for collaboration, may guide other health care providers to consider how they can individually and collectively maintain a patient-focus and use communication strategies that are aimed at achieving greater reciprocity within their health care team. Ultimately, understanding the characteristics of each developmental stage, the importance of co-location, patient-focus, and communication strategies and the need to be flexible may position health care providers from a variety of professional backgrounds to successfully navigate the journey of developing relationships that may provide improved patient care.
